# Interpretation of somatic *POLE* mutations in endometrial carcinoma

**DOI:** 10.1002/path.5372

**Published:** 2020-01-29

**Authors:** Alicia León‐Castillo, Heidi Britton, Melissa K McConechy, Jessica N McAlpine, Remi Nout, Stefan Kommoss, Sara Y Brucker, Joseph W Carlson, Elisabeth Epstein, Tilman T Rau, Tjalling Bosse, David N Church, C Blake Gilks

**Affiliations:** ^1^ Department of Pathology Leiden University Medical Center Leiden The Netherlands; ^2^ Faculty of Medicine University of British Columbia Vancouver Canada; ^3^ Contextual Genomics Inc Vancouver Canada; ^4^ Department of Gynaecology, Division of Gynaecologic Oncology University of British Columbia and BC Cancer Agency Vancouver Canada; ^5^ Department of Medical and Radiation Oncology Leiden University Medical Center Leiden The Netherlands; ^6^ Department of Women's Health Tübingen University Hospital Tübingen Germany; ^7^ Department of Oncology–Pathology, Karolinska Institutet, and Department of Pathology and Cytology Karolinska University Hospital Stockholm Sweden; ^8^ Department of Clinical Science and Education, Karolinska Institutet, and Department of Obstetrics and Gynaecology, Södersjukhuset Stockholm Sweden; ^9^ Institute of Pathology University of Bern Bern Switzerland; ^10^ Wellcome Centre for Human Genetics University of Oxford Oxford UK; ^11^ National Institute for Health Research (NIHR) Oxford Biomedical Research Centre Oxford University Hospitals NHS Foundation Trust, John Radcliffe Hospital Oxford UK; ^12^ Department of Pathology and Laboratory Medicine University of British Columbia and Vancouver General Hospital Vancouver Canada

**Keywords:** POLE, molecular classification, endometrial cancer

## Abstract

Pathogenic somatic missense mutations within the DNA polymerase epsilon (*POLE*) exonuclease domain define the important subtype of ultramutated tumours (‘*POLE*‐ultramutated’) within the novel molecular classification of endometrial carcinoma (EC). However, clinical implementation of this classifier requires systematic evaluation of the pathogenicity of *POLE* mutations. To address this, we examined base changes, tumour mutational burden (TMB), DNA microsatellite instability (MSI) status, *POLE* variant frequency, and the results from six *in silico* tools on 82 ECs with whole‐exome sequencing from The Cancer Genome Atlas (TCGA). Of these, 41 had one of five known pathogenic *POLE* exonuclease domain mutations (EDM) and showed characteristic genomic alterations: C>A substitution > 20%, T>G substitutions > 4%, C>G substitutions < 0.6%, indels < 5%, TMB > 100 mut/Mb. A scoring system to assess these alterations (POLE‐score) was developed; based on their scores, 7/18 (39%) additional tumours with EDM were classified as *POLE*‐ultramutated ECs, and the six *POLE* mutations present in these tumours were considered pathogenic. Only 1/23 (4%) tumours with non‐EDM showed these genomic alterations, indicating that a large majority of mutations outside the exonuclease domain are not pathogenic. The infrequent combination of MSI‐H with *POLE* EDM led us to investigate the clinical significance of this association. Tumours with pathogenic *POLE* EDM co‐existent with MSI‐H showed genomic alterations characteristic of *POLE*‐ultramutated ECs. In a pooled analysis of 3361 ECs, 13 ECs with DNA mismatch repair deficiency (MMRd)/MSI‐H and a pathogenic *POLE* EDM had a 5‐year recurrence‐free survival (RFS) of 92.3%, comparable to previously reported *POLE‐*ultramutated ECs. Additionally, 14 cases with non‐pathogenic *POLE* EDM and MMRd/MSI‐H had a 5‐year RFS of 76.2%, similar to MMRd/MSI‐H, *POLE* wild‐type ECs, suggesting that these should be categorised as MMRd, rather than *POLE*‐ultramutated ECs for prognostication. This work provides guidance on classification of ECs with *POLE* mutations, facilitating implementation of *POLE* testing in routine clinical care. © 2019 The Authors. *The Journal of Pathology* published by John Wiley & Sons Ltd on behalf of Pathological Society of Great Britain and Ireland.

## Introduction

Pathogenic somatic mutations in the exonuclease domain of the replicative DNA polymerase epsilon (*POLE*) define a subgroup of endometrial cancers (ECs) with ultramutation (frequently ≥ 100 mutations/Mb), characteristic mutation signature (COSMIC signature 10) 1, enhanced immune response 2, 3, and excellent clinical outcome 4, 5, 6, 7. ‘*POLE* ultramutated’ EC (*POLE*mut EC) has therefore been proposed as a distinct clinical entity that can be diagnosed in the presence of a pathogenic *POLE* exonuclease domain mutation (EDM) 8. For the five most common *POLE* mutations (P286R, V411L, S297F, A456P, and S459F), pathogenicity (in this sense meaning causal for tumour ultramutation) has been confirmed 4, 5, 6, 9, 10, 11, 12, 13, 14, 15, 16, 17, 18, 19, 20, 21, 22, 23, 24, 25; however, the classification of other, less frequent *POLE* variants is currently challenging. This is becoming an urgent problem, as *POLE* sequencing for molecular EC classification is rapidly entering clinical practice.

Previous work has shown that ECs with a pathogenic *POLE* EDM typically display characteristic genomic alterations, with a high prevalence of C>A substitutions, frequently exceeding 20%; a low proportion of small insertion and deletion mutations (indels); and an extremely high tumour mutational burden (TMB; > 100 mut/Mb) 12, 26. In the pivotal 2013 EC study from The Cancer Genome Atlas (TCGA), all 17 tumours classified as ultramutated had a *POLE* EDM, including recurrent P286R and V411L substitutions (eight and five cases, respectively), and one case each of S297F, A456P, M444K, and L424I substitutions 7. Interestingly, 10 of 231 non‐ultramutated ECs in this study also had a *POLE* mutation either within or outside the exonuclease domain. Following the TCGA report, further studies have confirmed the prevalence of the five pathogenic mutations listed above and identified additional variants of uncertain pathogenicity. The parameters by which to evaluate the latter are ill defined, and thus classification of such cases is challenging, particularly in the absence of whole‐exome or whole‐genome sequencing (WES/WGS). In order to facilitate the classification of ECs in clinical practice, we aimed to develop a scoring system to estimate the pathogenicity of novel *POLE* mutations based on the presence or absence of genomic alterations associated with known pathogenic *POLE* mutations. We also sought to provide pragmatic guidelines for the interpretation of *POLE* variants in cases analysed by targeted *POLE* sequencing where such comprehensive genomic data are unavailable, being mindful that the designation of a tumour as *POLE*‐ultramutated EC may lead to withholding treatment, given the very favourable prognosis of this EC molecular subtype, so that a conservative approach to diagnosis is warranted.

## Materials and methods

### Data extraction TCGA EC cohort

To analyse the base change proportions of the TCGA cohort of ECs (*n* = 530), we downloaded the MAF files [using Mutect for point somatic mutation call as well as small insertions and deletions (indels)] from Genome Data Commons (https://portal.gdc.cancer.gov/; accessed 27 February 2019). We used somatic called coding variants [single nucleotide substitutions (SNV), including synonymous mutations, and indels] as mutation count. To calculate tumour mutational burden (TMB), we used 38 Mb as the estimate of the exome size. Microsatellite status, as defined by the Bethesda Protocol classification 27, was obtained from the Genome Data Analysis Center (GDAC) database (https://gdac.broadinstitute.org/; accessed 30 October 2018).

COSMIC signatures from all 530 TCGA ECs were obtained from mSignatureDB (http://tardis.cgu.edu.tw/msignaturedb/; accessed 22 October 2019) 28, 29.

### Recurrence of somatic *POLE* mutations in EC and pan‐cancer

We searched for each somatic *POLE* mutation in the complete TCGA (Genome Data Commons) catalogues and COSMIC (https://cancer.sanger.ac.uk/cosmic, accessed 10 January 2019), annotating their recurrence on all cancer types (pan‐cancer) and exclusively within ECs (supplementary material, Table S1). Recurrent mutations were defined as those present in two or more cancer samples in the COSMIC and TCGA databases combined (cases present in both databases were counted only once). A mutation was considered non‐recurrent if it was found only once.

### *In silico* prediction tools

To evaluate the functional status of somatic *POLE* mutations, we used six widely‐used *in silico* tools: SIFT 30, PROVEAN 31, PolyPhen‐2 32, PANTHER 33, SNAP2 34, and the meta predictor REVEL 35. SIFT is a multi‐step algorithm using sequence‐based predictive features to predict the effect of single‐nucleotide polymorphisms (SNPs) 30. PROVEAN extends this approach, additionally incorporating analysis of in‐frame insertions, deletions, and multiple substitutions 31. PolyPhen‐2 implements sequence‐based and structure‐based predictive features and compares wild‐type and mutant allele through a decision tree 32; ‘possibly damaging’ results were interpreted as benign. PANTHER is based on protein sequence, using a metric based on evolutionary conservation on direct ancestors of the organism 33; ‘possibly damaging’ and ‘probably benign’ results were interpreted as benign. SNAP2 is a neural network‐based classifier that uses sequence and structural‐based data as inputs 34. REVEL is an ensemble method based on 13 individual tools 35; scores below 0.5 were considered benign.

### Somatic *POLE* mutations reported in ECs and not detected in TCGA cases

A review of the literature was undertaken, to the end of 2018, to identify ECs in which *POLE* had been sequenced and the mutations published 6, 7, 9, 10, 11, 12, 13, 14, 15, 17, 18, 19, 20, 21, 22, 24, 25. All literature contributing entries into the COSMIC database (POLE + endometrium) were reviewed; in addition, searches in PubMed and Web of Science were undertaken with the keywords ‘POLE + Endometrial + Carcinoma’ and ‘POLE + Endometrial + Cancer’, noting that ‘POLϵ’ is interpreted as ‘POLE’ in these resources.

### DNA mismatch repair‐deficient/microsatellite‐unstable, *POLE* exonuclease domain‐mutated endometrial cancer cohort

Tumours with concomitant mismatch repair deficiency (MMRd) and somatic *POLE* EDM, and clinical follow‐up were identified from a pooled cohort of 2988 molecularly profiled ECs across ten participating institutes (a detailed description can be found in León‐Castillo *et al* 36). Informed consent and ethical approvals were obtained according to local protocols in each participating centre. These tumours were combined with five tumours with concomitant microsatellite instability and *POLE* EDM from the 2013 TCGA EC cohort 7 for survival analysis.

### Statistical analysis

Nominal variables were compared by χ^2^ statistics or Fisher's exact test and ordinal variables using the Mann–Whitney test. All statistical tests were two‐sided and statistical significance was accepted at *p* < 0.05. We generated Kaplan–Meier curves for recurrence‐free survival (RFS) and overall survival (OS), and differences were tested by the log‐rank test. The median follow‐up was estimated by the reverse Kaplan–Meier method.

## Results

### Genomic characteristics of endometrial cancers with somatic *POLE* mutations in the complete TCGA cohort

To elucidate which genomic alterations best define pathogenic somatic *POLE* mutations (which we use in this context to mean very likely causal for tumour ultramutation), we used data from 530 ECs profiled by TCGA, including those reported in the 2013 publication 7. This included 82 tumours with a somatic *POLE* mutation, of which 59 (72%) were located within the exonuclease domain and 23 (28%) outside the exonuclease domain. The 59 exonuclease domain mutations comprised 21 unique variants, the five most common of which (P286R, 21 cases; V411L, 13 cases; S297F, 3 cases; A456P, 2 cases; and S459F, 2 cases) were classified as pathogenic based on previous reports 7, 8, 26 and designated as ‘hotspot’ *POLE* mutations for the purpose of this study (Table 1).

**Table 1:** *POLE* variants in TCGA EC

Protein change	No. of cases	Nucleotide substitution	Exon	MSI‐H cases (%)	Mutation recurrence in EC	Mutation recurrence pan‐cancer	No. of ‘benign’ results by *in silico* tools	POLE‐score	EDM	Signature 10 contribution
**P286R**	21	c.857C>G	9	1 (4.8)	Recurrent	Recurrent	0	5–6	Y	0.225–0.978
**V411L**	13	c.1231G>T/C	13	1 (7.7)	Recurrent	Recurrent	1	4–6	Y	0.000–0.751
**S297F**	3	c.890C>T	9	2 (66.7)	Recurrent	Recurrent	0	5–6	Y	0.123–0.611
**S459F**	2	c.1376C>T	14	0 (0)	Recurrent	Recurrent	1	5–6	Y	0.940–0.955
**A456P**	2	c.1366G>C	14	0 (0)	Recurrent	Recurrent	0	5–6	Y	0.277–0.837
**F367S**	2	c.1100T>C	11	2 (100)	Recurrent	Recurrent	0	6	Y	0.095–0.100
**L424I**	2	c.1270C>A	13	2 (100)	Recurrent	Recurrent	1	5 or 3	Y	0.000–0.000
**M295R**	1	c.884T>G	9	1 (100)	Recurrent	Recurrent	0	6	Y	0.785
**P436R**	1	c.1307C>G	13	0 (0)	Recurrent	Recurrent	0	6	Y	0.230
**M444K**	1	c.1331T>A	13	0 (0)	Recurrent	Recurrent	0	5	Y	1.000
R705W	1	c.2113C>T	19	0 (0)	Novel	Novel	1	5	N	0.821
**D368Y**	1	c.1102G>T	11	1 (100)	Novel	Recurrent	0	4	Y	0.042
M1754V	1	c.5260A>G	39	1 (100)	Novel	Novel	5	3	N	0.000
K1070N	1	c.3210G>T	26	1 (100)	Novel	Novel	1	3	N	0.000
L424V	1	c.1270C>G	13	0 (0)	Recurrent	Recurrent	0	3	Y	0.529
A428T	1	c.1282G>A	13	0 (0)	Novel	Novel	5	3	Y	0.000
R742H	1	c.2225G>A	20	1 (100)	Novel	Recurrent	1	3	N	0.018
Q1335*	1	c.4003C>T	30	1 (100)	Novel	Novel	NA	3	N	0.000
T278M	1	c.833C>T	9	1 (100)	Recurrent	Recurrent	0	3	Y	0.000
A465V	1	c.1394C>T	14	1 (100)	Recurrent	Recurrent	0	3	Y	0.000
S461L	1	c.1382C>T	14	1 (100)	Novel	Novel	0	2	Y	0.000
R114*	1	c.340C>T	5	1 (100)	Recurrent	Recurrent	NA	2	N	0.000
F990C	1	c.2969T>G	25	0 (0)	Novel	Novel	0	1	N	0.000
W1824C	1	c.5472G>T	40	0 (0)	Novel	Novel	0	1	N	0.000
E396G	1	c.1187A>G	12	1 (100)	Recurrent	Recurrent	2	1	Y	0.000
A1140T	1	c.3418G>A	28	1 (100)	Novel	Recurrent	5	1	N	0.000
Y1889C	1	c.5666A > G	41	1 (100)	Novel	Novel	0	1	N	0.000
A781S	1	c.2341G>T	21	1 (100)	Novel	Novel	6	1	N	0.000
R34C	1	c.100C>T	2	0 (0)	Recurrent	Recurrent	1	1	N	0.000
E1461V	1	c.4382A>T	34	1 (100)	Novel	Novel	5	1	N	0.000
R976S	1	c.2926C > A	25	0 (0)	Novel	Novel	1	0	N	0.000
V2025M	1	c.6073G>A	44	1 (100)	Novel	Novel	6	0	N	0.000
A566T	1	c.1696G>A	16	1 (100)	Novel	Novel	2	0	N	0.000
R1386Q	1	c.4157G>A	33	0 (0)	Novel	Novel	2	0	N	0.022
D368*	1	c.1101dupT	11	1 (100)	Novel	Novel	NA	0	Y	0.011
R1321K	1	c.3962G>A	31	1 (100)	Novel	Novel	5	0	N	0.000
Q1049H	1	c.3147G>T	26	1 (100)	Novel	Novel	2	0	N	0.000
R764M	1	c.2291G>T	20	1 (100)	Novel	Novel	0	0	N	0.000
E1698D	1	c.5094G > T	38	1 (100)	Novel	Novel	1	0	N	0.000
A1010T	1	c.3028G>A	25	1 (100)	Novel	Novel	1	0	N	0.000
C402R	1	c.1204T>C	12	1 (100)	Novel	Novel	3	0	Y	0.000
T906I	1	c.2717C>T	24	1 (100)	Novel	Novel	0	0	N	0.000
Q352H	1	c.1056G>T	11	1 (100)	Novel	Novel	4	0	Y	0.000
Q453R	1	c.1358A>G	13	1 (100)	Novel	Novel	3	0	Y	0.000

NA, not assessable. Pathogenic mutations in the exonuclease domain are in bold. Y = yes; N = no.

**Figure 1:**
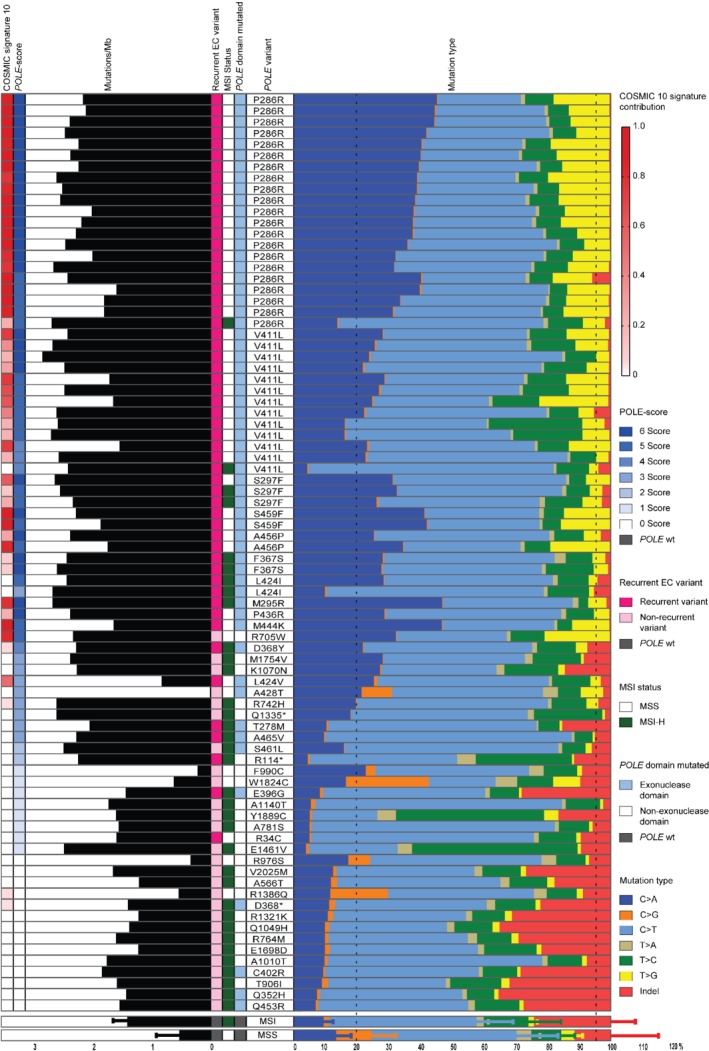
Mutational features of EC with *POLE* variants in the TCGA. The colour scheme for the mutation type is on the right of the histogram. Cases are grouped by mutations, with the most frequent *POLE* mutations in first place. The COSMIC 10 signature contribution, the points obtained in the *POLE* pathogenicity score (POLE‐score), the recurrence of the variant in EC, microsatellite instability (MSI) status, and *POLE* domain mutated are colour‐coded (legend on the right of the histogram). Below are the cases without *POLE* mutations; two rows depict the median plus standard deviation of the base change proportions and tumour mutation burden (TMB) of MSI‐H and MSS ECs without a *POLE* mutation in the TCGA.

The location of each *POLE* mutation (exonuclease domain versus non‐exonuclease domain), its recurrent or non‐recurrent status in EC in the TCGA and COSMIC databases, and genomic characteristics are shown for all cases in Figure 1. As previously reported 1, 7, 12, 26, the five hotspot *POLE* mutations were reliably associated with elevated TMB (median = 268 mut/Mb), which exceeded 100 mut/Mb (typically used to define ultramutation) in most tumours (33/41). Interestingly, TMB varied between different hotspot mutations (range 37.5–791.9 mut/Mb), and among tumours with identical hotspot mutations (e.g. P286R: 41.9–550.1 mut/Mb). *POLE* hotspot‐mutant ECs typically displayed a high proportion of C>A substitutions (median 32.5, > 20% in 37/41 tumours) and T>G substitutions (median 12.8%), whereas the proportion of C>G substitutions (median 0.3%) and indels (median 0.5%) was small. For comparison, 321 microsatellite‐stable (MSS), *POLE* wild‐type ECs all had TMB < 100 mut/Mb (median 2.1), a lower C>A proportion (median 13.5%) and T>G proportion (median 3.9%), and a higher C>G proportion (median 8.9%) and indel proportion (median 7.4%) (Table 2). We defined tumours with *POLE* hotspot mutations as a set of ‘true positives’, for subsequent identification of genomic alterations associated with pathogenic *POLE* mutations (Table 2).

**Table 2:** Tumour mutation burden and SNV/indel by *POLE* mutation location and tumour MSI status in TCGA endometrial cancers

	ECs with hotspot *POLE* mutations			ECs with non‐hotspot *POLE* EDMs			ECs with *POLE* non‐EDMs				
	Total	MSS	MSI	Total	MSS	MSI	Total	MSS	MSI	MSI‐*POLE*wt ECs	MSS‐*POLE*wt ECs
	*n* = 41	*n* = 37	*n* = 4	*n* = 18	4,0	*n* = 14	*n* = 23	*n* = 6	*n* = 17	*n* = 127	*n* = 321
Tumour mutational burden											
Median (range)	268.0 (37.5–791.9)	262.8 (37.5–791.9)	339.0 (237.7–550.1)	164.4 (1.1–530.4)	27.3 (1.1–262.9)	207.1 (26.9–530.4)	42.8 (1.7–452.9)	4 (1.7–236.2)	48.5 (17.4–452.9)	21.5 (0.0–150.5)	2.1 (0.3–59.0)
≥ 100 mut/Mb (%)	33 (80.5)	29 (78.4)	4 (100)	10 (55.6)	1 (25)	9 (64.3)	7 (30.4)	1 (16.7)	6 (35.3)	1 (0.8)	0 (0)
Percentage of C:G>A:T											
Median (range)	32.5 (4.3–45.2)	33.0 (16.0–45.2)	20.0 (4.3–32.5)	20.2 (6.9–46.9)	27.0 (21.4–46.7)	10.8 (6.9–46.9)	10.8 (3.9–32.3)	16.9 (5.0–32.3)	9.9 (3.9–28.1)	9.1 (0.0–23.2)	13.5 (2.8–27.6)
Proportion ≥ 20% (%)	37 (90.2)	35 (94.6)	2 (50)	9 (50)	4 (100)	5 (35.7)	4 (17.4)	2 (33.3)	2 (11.8)	1 (0.8)	25 (7.8)
Percentage of C:G>G:C											
Median (range)	0.3 (0.2–0.6)	0.3 (0.2–0.6)	0.3 (0.2–0.6)	0.5 (0.2–9.5)	0.7 (0.3–9.5)	0.5 (0.2–2.0)	1.0 (0.2–26.1)	5.0 (0.3–26.1)	0.9 (0.2–2.0)	1.5 (0.0–8.8)	8.9 (0.0–47.7)
Proportion < 0.6% (%)	37 (90.2)	34 (91.9)	3 (75)	11 (61.1)	2 (50)	9 (64.3)	6 (26.1)	1 (16.7)	5 (29.4)	5 (3.9)	2 (0.6)
Percentage of C:G>T:A											
Median (range)	43.6 (26.2–77.4)	40.7 (26.2–63.1)	58.3 (50.8–77.4)	52.1 (35.2–77.7)	50.9 (35.2–55.3)	52.1 (40.9–77.7)	45.9 (20.5–77.7)	47.2 (21.0–70.2)	44.6 (20.5–77.7)	46.0 (0.0–79.9)	47.9 (4.7–85.5)
Percentage of T:A>A:T											
Median (range)	1.1 (0.5–1.7)	1.1 (0.5–1.6)	1.4 (0.8–1.7)	1.5 (0.7–4.8)	1.3 (1.1–4.8)	1.5 (0.7–3.4)	2.4 (1.1–6.8)	4.3 (1.1–6.8)	2.3 (1.1–5.9)	1.9 (0.0–8.2)	4.5 (0.0–12.4)
Percentage of T:A>C:G											
Median (range)	8.9 (5.2–29.5)	8.4 (5.2–29.5)	10.7 (7.4–11.8)	8.9 (3.1–15.1)	7.9 (4.6–12.1)	9.5 (3.1–15.1)	11.8 (9.2–52.2)	10.7 (9.2–11.8)	12.1 (9.5–52.2)	11.7 (2.4–100.0)	9.3 (1.3–30.3)
Percentage of T:A>G:C											
Median (range)	12.8 (2.9–21.7)	13.0 (4.0–21.7)	5.1 (2.9–7.0)	2.3 (0.6–11.7)	6.0 (3.2–11.7)	1.6 (0.6–5.8)	1.6 (0.6–20.5)	1.8 (1.2–20.5)	1.6 (0.6–4.5)	1.4 (0.0–8.0)	3.9 (0.0–13.1)
Proportion ≥ 4% (%)	38 (92.7)	36 (97.3)	2 (50)	6 (33.3)	3 (75)	3 (21.4)	3 (13.0)	2 (33.3)	1 (5.9)	6 (4.7)	154 (48.0)
Percentage of small indels											
Median (range)	0.5 (0.2–6.0)	0.5 (0.2–6.0)	2.8 (1.8–4.0)	5.2 (0.4–35.5)	1.5 (0.4–3.2)	6.7 (0.9–35.5)	9.5 (0.4–35.1)	8.9 (0.4–9.7)	14.5 (1.9–35.1)	24.8 (0.0–40.2)	7.4 (0.0–80.9)
Proportion < 5% (%)	39 (95.1)	35 (94.6)	4 (100)	8 (80)	4 (100)	4 (28.6)	4 (17.4)	1 (16.7)	3 (17.6)	3 (2.4)	76 (23.7)

Of the 41 TCGA ECs with a somatic non‐hotspot *POLE* mutation, 18 were located within the exonuclease domain. Comparing these with the 23 tumours with non‐exonuclease domain mutations, non‐hotspot *POLE* exonuclease domain‐mutant ECs had a higher TMB (median 164.4 versus 42.8 mut/Mb) and C>A proportion (median 20.2% versus 10.8%), and a lower C>G proportion (median 0.5% versus 1.0%) and indel proportion (median 5.2% versus 9.5%) (Table 2).

MSI status was available for all TCGA ECs, of which 35/82 cases with somatic *POLE* mutations (42.7%) were MSI‐H. Comparison between ECs with hotspot mutations and non‐hotspot mutations within and outside the exonuclease domain revealed striking differences: only 4/41 (9.8%) of the TCGA ECs with one of the five hotspot mutations were MSI‐H, whereas 14/18 (78%) ECs with a non‐hotspot exonuclease domain mutation and 17/23 (74%) ECs with a non‐exonuclease domain mutation were MSI‐H (*p* < 0.0001). Analysis of the genomic architecture of these tumours revealed notable differences between groups. Tumours with hotspot *POLE* mutations and MSI had a high TMB (median TMB of 339.0 mut/Mb, > 100 mut/Mb in all four cases) and a high proportion of C>A and T>G substitutions (median 20.0% and 5.1%, respectively), with a low proportion of C>G substitutions (median 0.3%) and indels (median 2.8%) (Table 2). Tumours with non‐hotspot *POLE* EDM and MSI had a lower TMB (median 207.1 mut/Mb, > 100 mut/Mb in 9/14 cases) and C>A and T>G proportions (median 10.8% and 1.6%, respectively), a similar proportion of C>G substitutions (median 0.5%), and higher indel proportion (median 6.7%) (Table 2). These differences were greater in tumours with a *POLE* mutation outside the exonuclease domain and concomitant MSI, which had a median TMB of 48.5 mut/Mb (> 100 mut/Mb in 6/17 cases); C>A and T>G proportions of 9.9% and 1.6%, respectively; a C>G frequency of 0.9%; and a median indel proportion of 14.5%. For comparison, of 127 MSI‐H ECs without a *POLE* mutation (MSI‐H ECs), only one case had a TMB above 100 mut/MB (median 21.5) or a C>A proportion above 20% (median 9.1%); these cancers also had low T>G proportions (median 1.4%),a higher C>G proportion (median 1.5%,) and a high indel proportion (median 24.8%) (Table 2). Thus, the genomic characteristics of MSI‐H cancers with a *POLE* mutation outside the exonuclease domain are similar to those of MSI‐H tumours without a *POLE* mutation. Consequently, the frequency with which MSI co‐exists with *POLE* mutation varies by *POLE* mutation location and is reflected in differing genomic architecture – consistent with variable pathogenicity of *POLE* mutations.

These analyses confirm that ECs with one of the five somatic hotspot *POLE* EDMs carry characteristic genomic sequence alterations distinct from MSI‐H and MSS, *POLE*‐wild‐type EC. These genomic alterations are variably present in cases with non‐hotspot *POLE* EDM and are uncommon in ECs with *POLE* mutations outside the exonuclease domain. The variation in the genomic correlates of *POLE* mutations by their location is mirrored by variation in the prevalence of MSI in cancers carrying these mutations, and in differences in the genomic architecture of tumours harbouring both defects. Collectively, these data confirm that different *POLE* mutations vary in pathogenicity and underscore the need for its reliable estimation to ensure accurate patient classification.

### Establishing a pathogenicity score for somatic *POLE* mutations

Motivated by our preliminary analyses, we next used the TCGA WES data to develop a scoring system to assess the pathogenicity of *POLE* mutations (defined as the likelihood that they are associated with the characteristic ultramutated phenotype), using the hotspot *POLE* mutations as a truth set. Taking TMB and C>A, T>G, C>G, and indel proportions as the most discriminating genomic alterations for these pathogenic mutations, and building on previous work 26, we developed a pragmatic scoring system in which tumours scored 1 point for each of the following: TMB > 100 mut/Mb; C>A ≥ 20%; T>G ≥ 4%; C>G ≤ 0.6%; and indels ≤ 5%. All 41 TCGA ECs with a hotspot *POLE* mutation scored 3–5 points, while 13/41 (31.7%) ECs with a non‐hotspot *POLE* mutation scored ≥ 3 points, including 8/18 with exonuclease domain mutations, while 19/23 tumours with *POLE* mutations outside the exonuclease domain had scores ≤ 2, the exceptions being three tumours with score 3 (each of which had likely pathogenic mutations in *POLD1*: D316G, S478N, and L606M) and one scoring 5 points with a *POLE* R705W mutation. We therefore chose to focus on mutations in the exonuclease domain, given the infrequent association of non‐exonuclease domain mutations with genomic alterations associated with the ultramutated phenotype.

**Figure 2:**
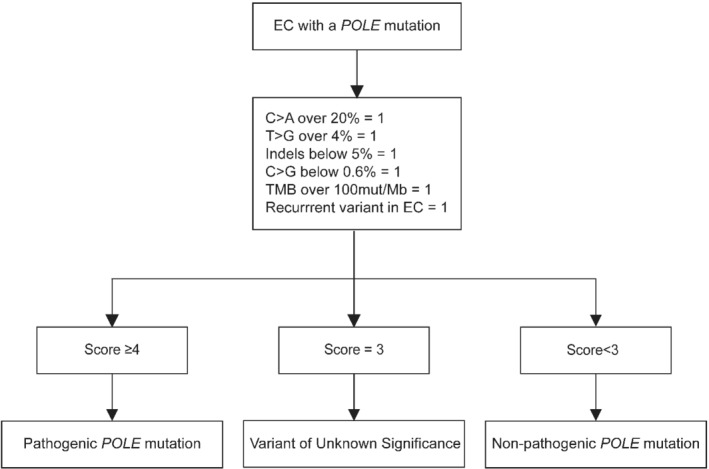
*POLE* genomic alteration score (POLE‐score). Diagnostic scoring system based on mutation type proportion and TMB of the five hotspot *POLE* mutations, as well as the variant recurrence.

To further refine this scoring system, we considered whether *POLE* mutations were recurrent in ECs within the COSMIC or TCGA databases, as recurrent mutations are more likely to be pathogenic (that is, causal of tumour ultramutation) 37. Forty‐eight out of 54 (88.9%) ECs scoring ≥ 3 points had a recurrent *POLE* mutation (including hotspot mutations), compared with 7/28 (25%) tumours scoring ≤ 2 points (*p* ≤ 0.001, χ^2^ statistics). Restricting the analysis to non‐hotspot *POLE* EDM, 7/8 (87.5%) tumours scoring ≥ 3 points had a recurrent mutation versus 5/10 (50%) scoring ≤ 2 points (*p* = 0.152, Fisher's exact test). Based on these results, ‘recurrence’ was incorporated into the final scoring system (Figure 2), which we termed the ‘POLE pathogenicity score’ (POLE‐score) (Table 1 and Figure 1).

To define a cut‐off for pathogenicity, we applied the POLE‐score on hotspot *POLE*‐mutant, non‐hotspot *POLE* EDM and control *POLE* wild‐type ECs (MSS and MSI‐H) in the TCGA cohort. Thirty‐eight of 41 (92.7%) ECs with a hotspot *POLE* EDM had a POLE‐score of ≥ 5 points (Figure 1). The remaining three tumours, all of which harboured a V411L mutation, scored 4 points. In contrast, of the 18 tumours with a non‐hotspot *POLE* EDM, seven scored ≥ 4 points (all of which carried mutations recurrent in the TCGA or COSMIC EC databases: F367S, L424I, M295R, P436R, M444K, D368Y), five scored 3 points (four of which carried recurrent mutations: A465V, L424V, T278M, L424I; one with a non‐recurrent A428T substitution), and six scored ≤ 2 points (one of which had a recurrent mutation). For comparison, all 321 MSS, *POLE* wild‐type ECs scored ≤ 3 points and all 127 MSI‐H *POLE* wild‐type ECs scored ≤ 2.

Based on these data, we used a POLE‐score of ≥ 4 points to define pathogenicity of *POLE* mutations in EC. When applying this cut‐off, 48 ECs in the TCGA are classified as having pathogenic *POLE* EDM (all 41 cases with hotspot mutations and seven with non‐hotspot variants), comprising 11 unique mutations, all of which are recurrent in TCGA/COSMIC (Table 3). ECs with a POLE‐score ≤ 2 were classified as having non‐pathogenic *POLE* EDM, based on the absence of genomic alterations associated with ultramutated phenotype. Cancers with a score of 3 (A465V, L424V, T278M, and A428T) were classified as having a variant of uncertain significance.

**Table 3:** Pathogenic *POLE* EDM based on POLE‐score

Protein change	Nucleotide substitution
P286R	c.857C>G
V411L	c.1231G>T/C
S297F	c.890C>T
S459F	c.1376C>T
A456P	c.1366G>C
F367S	c.1100T>C
L424I	c.1270C>A
M295R	c.884T>G
P436R	c.1307C>G
M444K	c.1331T>A
D368Y	c.1102G>T

To validate the POLE‐score, we noted the contribution of COSMIC signature 10 in ECs with a *POLE* EDM EC with a POLE‐score ≥ 4 points: signature 10 was present in 46 ECs (95.8%) and completely absent in two ECs (L424I and V411L) (mean 0.623, range 0.000–1.000). The contribution of signature 10 in ECs with one of the five hotspot *POLE* EDMs ranged from 0.978 to 0.123. Only in one (20.0%) EC with a *POLE* EDM classified as VUS (L424V) and one (16.7%) EC with a *POLE* EDM classified as non‐pathogenic (D368*) was activity of COSMIC signature 10 identified (mean contribution 0.106, range 0.00–0.529 and mean 0.002, range 0.000–0.011, respectively for each group). In comparison, COSMIC signature 10 was identified in 11 (8.7%) MSI‐*POLE*wt and 96 (29.9%) MSS‐*POLE*wt ECs (mean signature 10 contribution 0.002, range 0.000–0.048, and mean 0.017, range 0.000–0.218, respectively).

### Relationship between pathogenicity of somatic *POLE* mutations, microsatellite instability, and clinical outcome

The co‐existence of *POLE* mutations and MMRd/MSI in EC 26, 38 and the variation in its prevalence by *POLE* mutation location raise important questions about which is the initial, presumably dominant factor determining tumour phenotype and clinical outcome. To further investigate this, we used the POLE‐score to stratify TCGA cases into predicted pathogenic and non‐pathogenic *POLE* mutations using a score of ≥ 4. Nine of 49 (18.4%) ECs with a predicted pathogenic *POLE* mutation (including four known hotspot mutations) were MSI‐H, compared with 26/33 (78.8%) tumours with a predicted non‐pathogenic mutation (*p* ≤ 0.0001, χ^2^ statistic). Restricting the analysis to tumours with *POLE* EDM, 9/48 (18.8%) cases with a predicted pathogenic EDM (including hotspot mutations) were MSI‐H, as opposed to 9/11 (81.8%) with a predicted non‐pathogenic EDM (*p* ≤ 0.0001, Fisher's exact test). Interestingly, further stratification suggested a similar variation between likely pathogenic *POLE* mutations, as only 2/34 ECs with a P286R or V411L mutation were MSI‐H, compared with 7/14 ECs with one of the other nine predicted pathogenic mutations (*p* = 0.0012). Thus, *POLE* mutations co‐existent with MSI in EC are more likely to be non‐exonuclease, non‐pathogenic mutations, though this is not universally the case.

**Figure 3:**
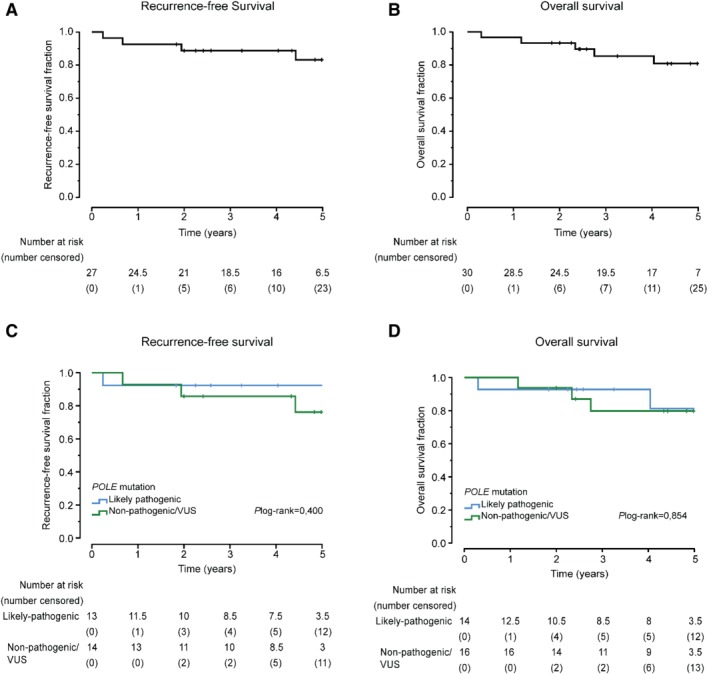
Clinical outcome of MMRd–*POLE*mut ECs. Kaplan–Meier survival curves for RFS (A) and OS (B) of MMRd–*POLE*mut ECs. RFS and OS of MMRd–*POLE*mut ECs with a pathogenic *POLE* EDM (mutation present in Table 3) versus all other tumours MMRd–*POLE*mut (C and D).

To investigate the clinical outcome of *POLE* exonuclease domain‐mutant EC with concomitant MMRd, we identified 30 such patients from a pooled analysis of 3236 ECs (Table 4). Five‐year recurrence‐free survival (RFS) for this subgroup was 83.2%, with a 5‐year overall survival (OS) of 80.9% (Figure 3) (corresponding figures for 24 patients with stage I disease were 84.2% and 85.4%, respectively) (supplementary material, Figure S1), seemingly contrasting with the 5‐year RFS and OS of 92–100% previously reported for *POLE* exonuclease domain‐mutant EC 4, 5, 7. To clarify this, we stratified patients according to predicted pathogenic versus non‐pathogenic EDM using the POLE‐score and analysed their clinical outcome. For cases that lacked WES data and for which *POLE* EDM had not been previously described in the TCGA, we considered all mutations different to the ones present in Table 3 (mutations deemed pathogenic using the POLE‐score) as VUS. This revealed that the 13 cases with one of the 11 mutations classified as likely pathogenic by POLE‐score (Table 3) had a 5‐year RFS of 92.3%, while the corresponding value for the 14 patients with EDM classified as likely non‐pathogenic/VUS was 76.2% (*p* = 0.40, log‐rank test) (Figure 3). While the clinical behaviour of tumours with combined MMRd/MSI and *POLE* EDM may vary based on the pathogenicity of the latter, this difference was not statistically significant, possibly owing to insufficient power/small numbers of cases, and it is not possible to determine the prognosis of this subgroup with certainty at present.

**Table 4:** Clinicopathological features of MMRd–*POLE*mut ECs

	MMRd–*POLE*mut ECs
	*n* = 30 (100%)
Age, years	
Mean [range]	66.5 [27–87]
< 60	9 (30)
60–70	8 (26.7)
> 70	13 (43.3)
Stage	
IA	14 (46.6)
IB	10 (33.3)
II	3 (10)
III	4 (10)
IV	0 (0)
Histology	
Endometrioid	25 (83.3)
Serous	1 (3.3)
Mixed	2 (6.7)
Clear cell	3 (6.7)
Grade	
1–2	19 (63.3)
3	11 (36.7)
Myometrium invasion	
< 50%	13 (43.3)
> 50%	15 (56.7)
LVSI	
Absent	21 (70)
Present	4 (13.3)
Missing	5 (16.7)
Treatment	
None	7 (23.3)
Radiotherapy	10 (33.3)
Chemotherapy	1 (3.3)
Radiochemotherapy	5 (16.7)
Unknown	7 (23.3)
*POLE* mutations	
Pathogenic mutation	14 (46.7)
Non‐pathogenic mutation/variant of unknown significance	16 (53.3)

### Estimation of pathogenicity of somatic *POLE* mutations in the absence of exome or genome sequencing

Somatic mutation profiling in clinical practice is typically performed by targeted panel sequencing, rather than WES/WGS approaches at present. To develop a classification tool for ECs with somatic non‐hotspot *POLE* mutations that can be implemented using such data, we used mutation location, prior data, and *in silico* tools which estimate the probability that a mutation is damaging. We first noted that nearly all (> 95%) *POLE* mutations outside the exonuclease domain are classified as non‐pathogenic by POLE‐score. We next noted that in the case of exonuclease domain mutations reported in the TCGA, the POLE‐score can be used to estimate pathogenicity (Table 3). We finally noted that for *POLE* EDM not present in the TCGA, *in silico* prediction tools could be used to estimate pathogenicity. Further exploration of this revealed that 10/11 *POLE* EDMs classified as pathogenic by POLE‐score in the TCGA cases were universally predicted to be disruptive by six *in silico* tools, the exception being an L424I substitution predicted to be deleterious by five tools but benign by one (Table 1 and supplementary material, Table S2). However, of five *POLE* EDMs present in the TCGA but classified as non‐pathogenic by POLE‐score, one (S461L, POLE‐score 2) was predicted to be damaging by all six tools, while another variant (E396G, POLE‐score 1) was predicted to be damaging by four tools. Furthermore, of four mutations classified as uncertain pathogenicity with a POLE‐score of 3, three (A465V, L424V, T278M) were considered damaging by all *in silico* prediction tools, while the other (A428T) was considered benign by five out of six prediction tools. Thus, *in silico* tools appear sensitive but not specific for prediction of pathogenic *POLE* EDMs, in the sense of their being likely causal for tumour ultramutation.

To define the extent of the problem of ascribing pathogenicity to *POLE* mutations in clinical practice, we identified 296/3840 (7.7%) tumours with a somatic *POLE* mutation from EC cohorts other than TCGA 6, 7, 9, 10, 11, 12, 13, 14, 15, 17, 18, 19, 20, 21, 22, 24, 25 (Table 5 and supplementary material, Table S3). Of 296 non‐TCGA *POLE*‐mutant ECs reported in the literature, 15 had mutations outside the exonuclease domain, and 254 carried mutations in the exonuclease domain previously detected in the TCGA and classified by POLE‐scores as pathogenic (249 cases), of uncertain pathogenicity (four cases), or non‐pathogenic (one case). The remaining 27 cancers with *POLE* EDM could not be classified by POLE‐score because their genomic correlates are yet to be determined by WES. This represents 9.1% of all reported *POLE* mutations, or 0.7% of non‐TCGA molecularly subtyped ECs to date. Of these 27 unique *POLE* EDMs, one was predicted to be benign by most *in silico* tools, while the others were predicted to be damaging by four tools or more (Table 5). The greater negative predictive value than positive predictive value of these tools, noted above, suggests that benign predictions should carry more weight and that the former are non‐pathogenic mutations, while the latter should be regarded as of uncertain pathogenicity. Cases such as these could be prioritised for more comprehensive sequencing, such as WES to provide sufficient data to determine their POLE‐score.

**Table 5:** *POLE* EDMs in ECs not described previously in the TCGA

Protein change	No. of cases	Nucleotide substitution	Exon	MSI cases (%)	Mutation recurrent in EC	Mutation recurrent pan‐cancer	No. of benign results by *in silico* tools
A426V	1 (2.4)	c.1277C>T	13	Unknown	Recurrent	Recurrent	1
A456G	1 (2.4)	c.1367C>G	14	Unknown	Novel	Novel	1
A456V	1 (2.4)	c.1367C>T	14	0 (0)	Novel	Recurrent	0
D275V	1 (2.4)	c.824A>T	9	Unknown	Novel	Novel	0
D287E	2 (4.9)	c.861T>A/G	9	1 (50)	Novel	Novel	1
D462E	1 (2.4)	c.1386T>A/G	14	0 (0)	Novel	Novel	1
F367C	1 (2.4)	c.1100T>G	11	0 (0)	Novel	Novel	0
F367L	1 (2.4)	c.1101T>A/G	11	1 (100)	Novel	Novel	0
F367V	1 (2.4)	c.1099T>G	11	0 (0)	Novel	Novel	0
G364V	1 (2.4)	c.1091G>T	11	1 (100)	Novel	Novel	0
G388S	1 (2.4)	c.1162G>A	12	0 (0)	Novel	Novel	0
H342R	1 (2.4)	c.1025A>G	11	Unknown	Novel	Novel	5
L283F	1 (2.4)	c.847C>T	9	1 (100)	Novel	Novel	1
L424P	1 (2.4)	c.1271T>C	13	0 (0)	Novel	Novel	0
M299I	1 (2.4)	c.897G>A/C/T	9	0 (0)	Novel	Novel	0
M405I	1 (2.4)	c.1215G>A/C/T	12	0 (0)	Novel	Novel	2
P286L	1 (2.4)	c.857C>T	9	1 (100)	Novel	Recurrent	0
P286S	1 (2.4)	c.856C>T	9	0 (0)	Novel	Recurrent	0
P436S	2 (4.9)	c.1306C>T	13	1 (50)	Novel	Novel	0
P441L	1 (2.4)	c.1322C>T	13	0 (0)	Novel	Recurrent	1
R375Q	1 (2.4)	c.1124G>A	12	0 (0)	Novel	Novel	1
S297Y	1 (2.4)	c.889T>G	9	0 (0)	Novel	Recurrent	0
T323A	1 (2.4)	c.967A>G	10	1 (100)	Novel	Novel	1
T457M	1 (2.4)	c.1370C>T	14	0 (0)	Novel	Recurrent	2
T483I	1 (2.4)	c.1448C>T	14	0 (0)	Novel	Novel	0

### Recommendations for classification of somatic *POLE* mutations in clinical practice

Based on the analyses above, we developed a pragmatic tool to classify EC with somatic *POLE* mutations in clinical practice, shown in Table 6 39, 40. For cases with WES/WGS, POLE‐score and the presence or absence of MSI/MMRd can be used to stratify cases into *POLE*mut, MMRd, or one of the other two TCGA subgroups depending on p53 status (Singh *et al* 41). It is important to note that the presence of a *POLE* mutation alone is insufficient to classify tumours as ‘*POLE*mut’, and that classification of tumours with combined *POLE* mutation and MMRd/MSI depends on the POLE‐score (i.e. genomic correlates) of the *POLE* mutation. For cases without WES/WGS, POLE‐score can be used if the *POLE* mutation has previously been reported in the TCGA. Where this is not the case, *in silico* tools can be used to triage tumours for more comprehensive WES/WGS to permit calculation of POLE‐score and subsequent classification.

**Table 6:** Recommendations for the interpretation of somatic *POLE* mutations in ECs. Recommendations to classify ECs with *POLE* mutations with (A) POLE‐score available or (B) POLE‐score absent

A			
*POLE* mutation	Predicted pathogenicity	MSI/MMR status	Treatment recommendation
Exonuclease domain mutation	Pathogenic	MSS/MMRp	*POLE*mut EC
POLE‐score ≥ 4	Pathogenic	MSI/MMRd	*POLE*mut EC*
Exonuclease domain mutation	Non‐pathogenic	MSS/MMRp	*POLE*wt EC
POLE‐score < 4	Non‐pathogenic	MSI/MMRd	MMRd EC
Non‐exonuclease domain mutation	–	MSS/MMRp	NSMP/p53abn EC†
	–	MSI/MMRd	MMRd EC
If tumours‐only sequencing is performed, detection of L424V variant should prompt consideration of germline testing 39, 40.			

B			
*POLE* mutation	Predicted pathogenicity	MSI/MMR status	Treatment recommendation
Exonuclease domain mutation predicted to be pathogenic by ≥ 4 *in silico* prediction tools	VUS	MSS/MMRp	WES or NSMP/p53abn EC†, ‡
	VUS	MSI/MMRd	WES or MMRd EC‡
Exonuclease domain mutation predicted to be non‐pathogenic by > 1 *in silico* prediction tool	Non‐pathogenic	MSS/MMRp	NSMP/p53abn EC†
	Non‐pathogenic	MSI/MMRd	MMRd EC
Non‐exonuclease domain mutation	–	MSS/MMRp	NSMP/p53abn EC†
	–	MSI/MMRd	MMRd EC

If tumours‐only sequencing is performed, detection of L424V variant should prompt consideration of germline testing 39, 40.

* Treat as *POLE*mut EC (based on genomic alteration) independently of MMR status (insufficient data to suggest otherwise).

† p53 IHC should be performed to exclude a p53abn EC.

‡ Treat conservatively, i.e. as MMRd/NSMP or send for WES.

NSMP, no specific molecular profile; VUS, variant of unknown significance.

## Discussion

The development of pragmatic surrogate markers has accelerated the clinical implementation of molecular EC classification. The presence of a pathogenic *POLE* EDM is causal for ultramutated EC, a subtype associated with enhanced immune response 2, 42 and excellent clinical outcome 6, 7, 13. De‐escalating adjuvant treatment in these patients is currently under investigation in the randomised PORTEC4a trial. However, interpretation of *POLE* sequence variants is challenging due to lack of standardized criteria, other than for the most common ‘hotspot’ mutations for which pathogenicity is reliably established. We aimed to generate tools to estimate the pathogenicity of *POLE* mutations using WES data, and to guide the management of cases where comprehensive genomic profiling is not available.

Using cases with recurrent ‘hotspot’ *POLE* EDMs as a truth set, we identified their characteristic genomic correlates to generate a ‘POLE‐score’. In addition to correctly classifying all cases with *POLE* hotspot mutations in the TCGA cohort, it classified a further six *POLE* EDMs as likely pathogenic. Four exonuclease domain mutations had a POLE‐score of 3 and were classified as being of uncertain pathogenicity, while three cases with *POLE* mutations outside the exonuclease domain had a POLE‐score of 3 – all of which carried a plausibly pathogenic *POLD1* mutation that could explain the mutational spectrum 8. Intriguingly, a single case with a *POLE* mutation outside the exonuclease domain (R705W) was classified as pathogenic by POLE‐score. The location of the mutation within the catalytic domain, close to the polymerase active sites, may explain this mutational spectrum; however, the clinical significance of this is unclear at present.

Because POLE‐score relies on WES or WGS to estimate TMB and mutation proportions, it is unable to assign pathogenicity in the case of novel *POLE* mutations detected by targeted sequencing, where breadth is typically inadequate to estimate these parameters. Although this represents a potential challenge in clinical practice where targeted approaches are common, our pooled analysis suggests that this situation is uncommon – only 0.7% of ECs at the time of writing, a figure that will drop over the coming years as more WES/WGS data are accrued. We found that pathogenicity of such variants is not reliably predicted by *in silico* tools, which have low specificity. We suggest an approach to these tumours (outlined in Table 6), which may guide the use of additional sequencing (e.g. WES) to permit calculation of POLE‐score in these cases. Although WES remains relatively costly compared with targeted approaches, such outlay is modest against that of local or systemic therapy, and thus remains a possible approach for cases where a significant treatment decision hangs in the balance.

Our study confirms the complex relationship between *POLE* mutations and DNA mismatch repair deficiency/microsatellite instability in EC. Perhaps most straightforward are those with *POLE* mutations outside the exonuclease domain: these appear to be passengers secondary to the hypermutator phenotype and should be classified as MMRd ECs. Co‐existence of *POLE* EDM with MSI/MMRd is relatively uncommon, occurring in 3.4% cases in TCGA and 0.9% cases of molecularly subtyped tumours in our pooled series (this variation probably reflects a combination of targeted sequencing with enrichment for pathogenic *POLE* mutations in the latter cases). This group of tumours is heterogeneous. Those with *POLE* mutations predicted as pathogenic by POLE‐score and MSI had genomic architecture similar to *POLE* hotspot‐mutant/MSS tumours, supporting their classification as *POLE*mut EC. Those with *POLE* mutations predicted as non‐pathogenic by POLE‐score and MSI more closely resembled *POLE*‐wild‐type MSI cases, supporting their classification as MMRd EC. *POLE* EDM in combination with MMR loss causes a distinct mutational signature in EC (COSMIC signature 14) 1, 38 – the observation that this is not universal in cases with both defects supports the notion that these tumours are a heterogeneous group, where MSI/MMRd could be acquired after *POLE* EDM and vice versa, with differing impacts on prognosis. Interestingly, while data were limited, patients with combined pathogenic *POLE* EDM and MSI appeared to have a good clinical outcome in our pooled cohort (5‐year RFS 92.3%), though additional cases are required before this can be concluded.

In conclusion, our work provides guidance in the diagnostic interpretation of *POLE* mutations in EC in the presence and absence of WES data. Tumours with any of the 11 *POLE* EDMs identified in the TCGA and classified as pathogenic by POLE‐score should be classified as ‘*POLE* ultramutated’ EC, independently of MMRd/MSI status. For cases where a *POLE* EDM not present in the TCGA is identified and WES data are available, POLE‐score can be used for classification. In the absence of WES data, classification should be informed by the results of POLE‐score on mutations reported in the TCGA and classified in Table 3. *In silico* prediction tools have limited value but may be able to identify benign changes and triage cases for WES/WGS. The guidelines that we provide will evolve over time but will allow for almost all tumours encountered to be classified into a molecular subtype based on currently available information.

## Author contributions statement

AL and HB carried out experiments and analysed data. TB, CBG, DNC, and MM conceived experiments and analysed data. JNM, RN, SK, SYB, JWC, EE, and TTR analysed data. All the authors were involved in writing the paper and had final approval of the submitted and published versions.

## Supporting information


**Figure S1**. Clinical outcome of MMRd–*POLE*mut ECs

**Table S1**. *POLE* mutations reported in ECs in COSMIC or TCGA

**Table S2**. *In silico* tools results for *POLE* mutations found in ECs in TCGA

**Table S3**. *In silico* tools results for non‐hotspot *POLE* mutations found in EC cohorts published not present in TCGA
