# Supplementary figures and images for: Interpretation of somatic POLE mutations in endometrial carcinoma
Source: J Pathol. 2020 Jan 29;250(3):323–35. doi: 10.1002/path.5372 (PMC7065171; doi:10.1002/path.5372)

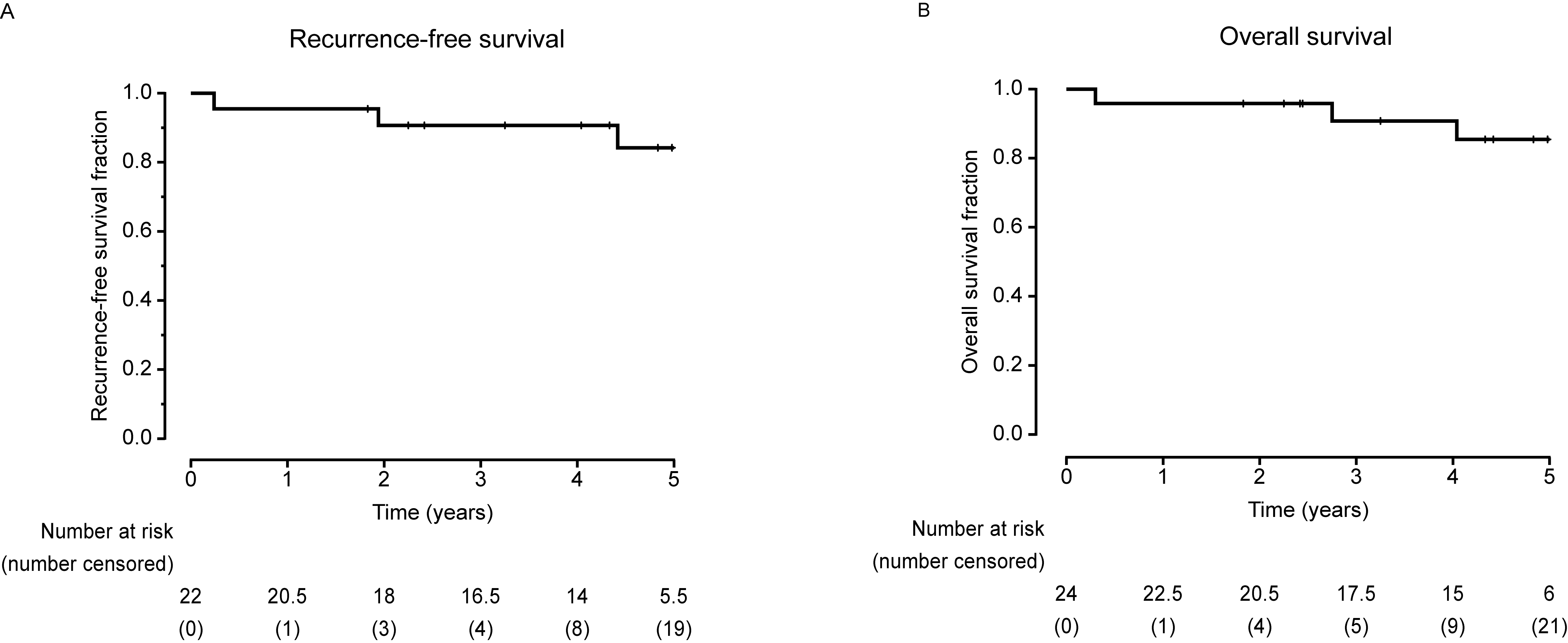

Supplement: Supplementary file 1 — Figure S1. Clinical outcome of MMRd–POLEmut ECs [file PATH-250-323-s001.tif]
